# First assessment of plague in terrestrial small mammals and fleas from Makira Natural Park and surroundings, North-eastern Madagascar

**DOI:** 10.1371/journal.pntd.0013710

**Published:** 2025-11-17

**Authors:** Beza Ramasindrazana, Mireille Harimalala, Fanohinjanaharinirina Rasoamalala, Cynthia Haingotiana Martin, Lanto Andrianarijaona Maminirina, Sylvie Claudia Raritahiry, Johan Michaux, Minoarisoa Rajerison, Julie Linchant, Pierre Walter, Daouda Kassié, Hélène Guis, Lucy Keatts, Ferran Jori

**Affiliations:** 1 Plague Unit, Institut Pasteur de Madagascar, Antananarivo, Madagascar; 2 Ecole Doctorale Sciences de la Vie et de l’Environnement, Université d’Antananarivo, Antananarivo, Madagascar; 3 Biological Resource Center, Institut Pasteur de Madagascar, Antananarivo, Madagascar; 4 Medical Entomology Unit, Institut Pasteur de Madagascar, Antananarivo, Madagascar; 5 Département d’Enseignement des Sciences et Médecine Vétérinaire, Université d'Antananarivo, Antananarivo, Madagascar; 6 Epidemiology and Clinical Research Unit, Institut Pasteur de Madagascar, Antananarivo, Madagascar; 7 French Agricultural Research Centre for International Development, UMR ASTRE, Antananarivo, Madagascar; 8 Laboratoire de génétique de la conservation, Institut de Botanique, Université de Liège, Liège, Belgium; 9 UMR ASTRE, Université de Montpellier, French International Research Centre for Agricultural Development, National Institute for Agricultural Research, Montpellier, France; 10 French International Research Centre for Agricultural Research, UMR ASTRE, Montpellier, France; 11 Wildlife Conservation Society (WCS), MaMaBay Program, Maroantsetra, Madagascar; 12 Wildlife Conservation Society (WCS), Health Program, Bronx, New York, United States of America; 13 Department of Zoology and Entomology, University of Pretoria, Pretoria, South Africa; University of Lucknow, INDIA

## Abstract

**Background:**

Plague, a zoonosis caused by *Yersinia pestis*, is endemic in Madagascar but knowledge on the epidemiological situation in the northern focus remains unclear. The aim of this study was to investigate the circulation of *Y. pestis* in terrestrial small mammals in north eastern Madagascar, where suspected plague outbreaks have been reported.

**Methods:**

Sampling of terrestrial small mammals and their fleas was carried out in 22 trapping sites within 9 localities of the two sectors (1 and 3) of Makira Natural Park (MNP) and surroundings, from 2020 to 2022. *Yersinia pestis* was investigated in terrestrial small mammal spleen samples and their fleas using bacteriological, serological and molecular methods.

**Results:**

A total of 614 terrestrial small mammals composed of eight species and 1,754 individual fleas were collected following 4,880 trap-nights. The black rat (*Rattus rattus*) represented the majority (87.8%) of the small mammal species caught. Flea infestation rate was higher in sector 3 compared to sector 1. In sector 3, *Xenopsylla brasiliensis*, a plague vector, represented 66.4% of fleas identified. Further, one plague seropositive *R. rattus* individual, captured inside a house, and one *Ctenocephalides felis* specimen, collected on another *R. rattus*, was positive on PCR in this sector.

**Discussion:**

Despite low detection rates, we confirmed the circulation of *Y. pestis* in our study area (one rat seropositive and one flea PCR positive) and highlight the risk of potential human transmission. Our results also suggest that *R. rattus* contributes to the maintenance and transmission of plague in MNP, as described for other areas in Madagascar. Further, these findings contribute to documentation of the known geographic distribution of the endemic plague vector *S. fonquerniei* and *X. brasiliensis*.

**Conclusion:**

The confirmation of the circulation of the *Y. pestis* through serological and molecular diagnostics in small mammals and fleas underscores the urgent need to assess awareness levels of risk factors and symptoms to monitor among local communities and health workers and ensure that trained rapid response teams are prepared to intervene promptly upon suspect case detection. The risk and epidemiology of plague circulation in remote rural areas of Madagascar remains insufficiently studied. Addressing this gap is crucial, as a more comprehensive understanding of the distribution and dynamics of the wild animal hosts, their vectors and host-vector interactions will enhance risk assessment and prevention for plague emergence and improve mitigation and early control of potential outbreaks.

## Author summary

Plague, a zoonosis caused by *Yersinia pestis*, is an endemic disease and major public health problem in Madagascar. Almost all of the available data are from the central highlands and little is known regarding the epidemiology of the disease in the northern region, despite suspect cases. The aim of this study was to investigate the circulation of *Y. pestis* in terrestrial small mammals and their fleas in north eastern Madagascar, where suspected plague outbreaks have been reported in the last decade. Sampling of terrestrial small mammals and their fleas was carried out in 22 trapping sites located in two different sectors (1 and 3) of Makira Natural Park (MNP) and its surroundings, from 2020 to 2022, to investigate the presence of *Y. pestis*. A total of 614 terrestrial small mammals composed of eight species and 1,754 flea individuals were collected. It is important to note that *Xenopsylla brasiliensis*, a known plague vector in Africa, represented about 65% of the fleas collected. After biological screening, one plague seropositive *R. rattus* individual, captured inside house as well as one PCR positive *Ctenocephalides felis* specimen collected on *R. rattus* were identified confirming the circulation of *Y. pestis* in the region and the importance of further investigation to understand the epidemiology of the disease in this plague focus and mitigate future outbreaks.

## Introduction

Plague, a flea-borne zoonosis caused by the Gram-negative bacterium, *Yersinia pestis,* cycles naturally in wild rodents and is primarily transmitted to humans by fleas. The bacterium has been responsible for three pandemics with the “black death” resulting in millions of deaths worldwide during the 14^th^ Century [1]. Plague can manifest itself in humans through different clinical presentations. Bubonic plague is the most common form and is acquired through infected flea bites. When untreated, this bubonic form may evolve into secondary pneumonic plague which can be transmitted very rapidly through direct human-to-human air-borne transmission [2]. Climate, insecticide resistance, and human behavior, amongst other factors, can influence the emergence or re-emergence of plague in a given region [3–6]. Clinical symptoms of plague are sufficiently characteristic to allow populations to launch an alert of suspicion and the implementation of a rapid and appropriate response, as long as awareness has been raised among local populations, health workers and the different stakeholders. Plague can be treated with antibiotics, but in remote rural areas in lower- and middle-income countries, access to treatment can remain a challenge.

To date, plague is still present in several countries of Asia, the Americas and Africa [7]. In Africa, 90% of notified cases are from Madagascar [7]. Most of these are bubonic cases from rural areas where plague is endemic and seasonal, with three specific foci identified: the northern part of the island, the Central Highlands and the sea port of Mahajanga [2,8,9]. In 2017, an outbreak with 2,414 suspected human cases was reported in Madagascar of which 23% probable cases were confirmed [10]. This outbreak had a very high socioeconomic burden in Madagascar and showed not only the weakness of our surveillance system but also the lack of information regarding plague epidemiology and distribution.

While introduced rodents (*Rattus rattus* and *R. norvegicus*) and the Asian shrew (*Suncus murinus*) represent the main reservoirs of plague in Madagascar [9], some studies have also reported the circulation of *Y. pestis* in certain species of the Tenrecidae family living in transition zones between forests and areas inhabited by humans through bacteriological, serological or molecular studies [2,11,12]. Two flea species are described as the main vectors, namely the cosmopolitan *Xenopsylla cheopis* and the endemic *Synopsyllus fonquerniei*. However, the vector status of other flea species requires investigation. The human flea *Pulex irritans* and the rat flea *Xenopsylla brasiliensis* are suspected to be involved in plague transmission [13,14].

The Sustainable Wildlife Management (SWM) Programme is a major international initiative that aims to improve wildlife conservation and food security by developing approaches to conserve wild animals and protect ecosystems, whilst at the same time improving the livelihoods of Indigenous People and rural communities who depend on these resources (https://www.swm-programme.info). In Madagascar, the project aims to support transition from the consumption of vulnerable and protected species to the sustainable use of non-protected and resilient wildlife species, such as tenrecs and bushpigs, and to develop fish and poultry farming to increase the supply of alternative proteins to replace wild meat consumption. One of the four outcomes targeted by the SWM Programme in Madagascar is to identify how the consumption of wild meat could be sustainable and a key component of this is consideration of potential health risks associated with meat consumption from wild species, including from zoonotic pathogens. The Covid-19 crisis, considered by many scientists to have originated from wild animal trade for food, illustrates the potential for catastrophic impacts resulting from emergence of a zoonotic disease.

Prior studies carried out in the Makira Natural Park (MNP) in North eastern Madagascar found that a large majority of households (91%) consumed insectivore species of the family Tenrecidae [15], the most common being *Setifer setosus* and *Tenrec ecaudatus* [16]. Various studies have highlighted the role of these small mammals as potential reservoirs of zoonotic pathogens such as leptospirosis [17–19], toxoplasmosis [20,21] and hantavirus infection [22–24]. It is known that, in addition to rodents, small insectivores such as tenrecs (*Tenrec ecaudatus* and *Setifer setosus*) may act as wild reservoirs of *Yersinia pestis* [2].

The Makira region, which spans part of Mandritsara, Befandriana and Maroantsetra districts, is located within the northern plague foci [13,25]. Recently, local populations living in the vicinity of the MNP reported the occurrence of disease outbreaks with symptoms suggestive of plague. However, these potential outbreaks were not confirmed by epidemiological investigations nor biological sample analyses, and further evidence of the circulation of plague in the area was lacking. In addition, an outbreak of human plague was reported in 2013–2014 in the District of Mandritsara (a locality close to sector 3 of MNP) [13]. The aim of this study was to investigate the potential circulation of *Yersinia pestis* in terrestrial small mammals and their fleas in the MNP and surroundings, as part of the northern plague foci in Madagascar, through bacteriological, serological and molecular analyses.

## Materials and methods

### Ethics statement

This study was carried out within the framework of the SWM Programme and obtained the authorization of the Animal Ethics Committee of the Institut Pasteur de Madagascar (N°002/2020/IPM/DS/CEA) as well as a research permit from the Ministry of the Environment and Sustainable Development (N°198/20/MEDD/SG/DGEF/DAPRNE and 185/22/MEDD/SG/DGGE/DAPRNE/SCBE.Re). The manager of MNP (WCS) was briefed on the study (objectives, modalities, constraints, benefits and risks, and right of refusal) prior to capture. All terrestrial small mammal individuals were trapped and handled in strict compliance with the guidelines accepted for the handling of wild animals [26] and the Directive 2010/63/EU of the European Parliament (http://eur-lex.europa.eu/Lex-UriServ/LexUriServ.do?uri=OJ:L:2010:276:0033:0079:EN:PDF).

#### Study area and sampling period.

The Makira Natural Park (MNP), created in 2012, is located in the north-east of Madagascar. It is one of the largest terrestrial protected areas in Madagascar with a surface around 372,470 hectares (approximately 3,700 km²) administratively divided into six sectors. It is home to the largest block of intact low- and medium-altitude dense rainforest in Madagascar. Since 2013, the Wildlife Conservation Society (WCS) has been the delegated manager by the Ministry in charge of protected areas. The Government and WCS have introduced community-based management of natural resources in the protection zone, or greenbelt, through Base Communities (COBAs for *COmmunautés de BAse*), legally registered community associations, each managed by a Management Committee (COGE for *COmité de GEstion*).

**Fig 1:**
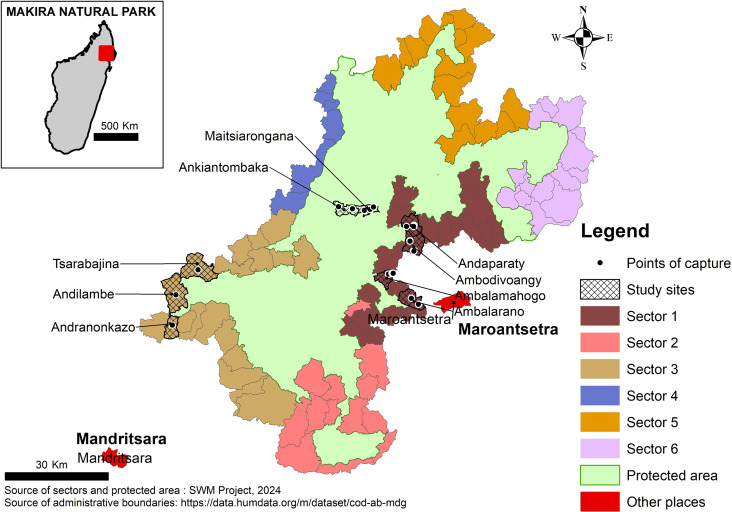
Location of the study sites in Makira Natural Park, Madagascar.

The SWM Programme targets specifically two of the six management sectors within MNP, sectors 1 and 3, chosen because (i) communities in these areas rely heavily on hunting for subsistence, (ii) these are biodiversity hotspots (iii) they are relatively more accessible than the other sectors and (iv) they are key sites to monitor the risk of zoonotic disease outbreaks. Indeed, the sector 1, located on the eastern part of MNP (Fig 1), is the most densely populated area of MNP and the sector 3, located on the most western part of MNP, is close to where suspected human plague were reported in 2013–2014. Study area comprised sites in the two sectors targeted by SWM Programme.

#### Sampling strategy.

Small mammal traps were deployed in three ecological habitats: (i) inhabited houses within villages, (ii) outside households following transect lines and (iii) when possible, in forested areas used for hunting by local people. The sampling strategy was constrained by field conditions, and in particular site accessibility. There is no road access to MNP. The closest villages of sector 1 can be reached by a four-hour boat ride from Maroantsetra, the district capital (Analanjirofo region), and then, from there, the villages or areas can be reached on foot. In sector 3, the closest villages from Mandritsara, the district capital (Sofia Region), can be reached by a three-hour ride by motorbike (dirt path), and again, from there other villages can be reached on foot.

In sector 1, sampling was carried out in 16 trapping sites in Maroantsetra district comprising 6 villages, 6 hunting zones (the hunting zones of the selected villages) and four buffer zones. The six villages included were Andaparaty, Ambalamahogo, Ambalarano, Ambodivoangy, Ankiatombaka and Maitsiarongana (Fig 1). The four buffer zones were located in the area surrounding the last two villages. In the sector 3, three locations in Mandritsara district were investigated, namely Andranonkazo, Andilambe and Tsarabanjina (Fig 1). In each of these three locations, two trapping sites were selected: one site in the village and one site in the neighboring forest, thus constituting a total of 6 sites. Field sampling was undertaken from October 2020 to April 2021 in sector 1, from 20 July to 12 August 2022 in sector 3. All of the sites surveyed are located in the lowland of Madagascar (altitude less than 800 m above sea level).

The sample size was estimated considering three hypotheses: (i) the sampling rate was smaller than 10% (sampling in a large population), (ii) trapping ensured a partial randomization and (iii) the occurrence of a hypothetical homogeneous mixing of individuals within the population in sampling sites. To be able to detect a threshold prevalence rate of 1% with a margin of error of 5% in a large population with a confidence interval of 95%, a sample size of at least 299 individuals was needed [27]. To achieve this sample size, considering a trapping rate of 10%, we estimated that a capture effort of at least 2,990 trap-nights was required. This defined the framework within which more arbitrary choices concerning the number of villages, hunting zones, buffer zones, and traps per site were made due to field constraints (in particular accessibility). All of the data collected associated with the present study are provided as a Supplementary material with the study (S1 Data).

#### Small mammal capture and sample collection.

Small mammals were captured using Sherman traps (H.B Sherman Trap, Inc., Tallahassee, FL; 23 cm long x 7.5 cm wide x 9 cm high) and wire-meshed live traps, either BTS (Besançon Technical Service, Besançon, France; 30 cm long x 10 cm wide x cm high) or Tomahawk flexible (100 × 80 × 80 cm and 81 × 23 × 23 cm) traps. The latter two traps are quite similar and allowed generally to capture bigger small mammals. All traps were placed directly on the ground, along transect lines, in locations likely to be frequented by small mammals (e.g., near burrows, runways, or food sources). Traps were set for four nights, left open 24 hours a day, and checked twice a day, in the morning and in the afternoon.

In sector 1, 50 live traps (25 Sherman and 25 Tomahawks) were set. In the villages, one trap (Tomahawk or Sherman) was set inside each house. In the 6 hunting zones and in the 4 buffer areas, the 50 traps (Tomahawks and Sherman) were set outside following a transect line with traps spaced by 10m apart. All traps were baited with peanut butter.

In sector 3, 75 small mammal traps encompassing 50 BTS traps (Tomahawks-like traps but made locally in Madagascar) and 25 Sherman traps were set in 3 sites both inside houses in villages (3 sites) and outdoors in the forested hunting zone surrounding the villages (3 sites). Traps were baited with dry fish, onions and peanut butter. In each house, one trap (BTS or Sherman) was set. In the forested sites in sector 3, the traps were distributed along transects by alternating two BTS traps followed by one Sherman trap. Two successive traps were spaced at about 10 m apart.

Due to logistic reasons and constraints, the number of traps set in both sectors was not the same. However, this allowed us to have an overview of the small mammal and fleas diversity present in both sectors in the northern portion of Madagascar.

All individuals identified as introduced species and a maximum of two individuals identified as endemic species captured in each site (as allowed by research permits) were euthanized by cervical dislocation before biological sampling. For these individuals, a blood sample was taken by heart puncture and preserved on dry filter paper. In addition, ectoparasites were collected, if any, and body measurements and sex determination were carried out. Finally, spleen samples were taken and stored in carry Blair transport medium at room temperature in the field prior to laboratory analysis within the Plague Unit at Institut Pasteur de Madagascar, in the capital city of Antananarivo.

If the number of individuals of endemic species captured exceeded two (the authorized number of euthanized individuals stipulated by the research permit), these small mammals were first anesthetized using Xylazine (5 mg/kg) and Ketamine (40 mg/kg) administered via the intraperitoneal route. A venipuncture (in caudal and/or femoral veins) was performed to collect a blood sample. Body measurements were taken to facilitate species identification according to published guidelines [28]. Atipamezole (0,1 mg/kg) was then used as an antidote to induce animal wake-up. When the individual had fully woken up after the effect of the antidote, the individual was released at the capture site.

## Flea collection

Fleas from small mammals were removed by brushing the fur of the animal using a soft brush, in both growing and opposite directions of fur growth inside a 30 cm deep basin. Fleas escaping from the host into the basin were collected using a homemade flea vacuum. They were preserved in 70% ethanol prior to laboratory identification and screenings. Species identification was performed under binocular magnifier and using available keys [29,30].

### Laboratory analysis

#### Bacteriological screening of *Y. pestis.*

Rapid Diagnostic Test (RDT) based on the principle of immunochromatography which was developed, produced, and used in the Plague Unit [31], was carried out on crushed spleen tissue solution. This was followed by bacteriological culture using Cefsulodin-Irgasan-Novobiocin (CIN) medium [32] according to the protocol of the Plague Unit - Central Laboratory for Plague at Institut Pasteur de Madagascar.

#### Molecular detection of *Y. pestis* in small mammals and fleas.

DNA extractions from spleen samples and one blood sample were done using Qiagen kit (Qiagen, Germany) and following the manufacturer’s protocol. Molecular screening was done using the algorithm used in the central laboratory for plague targeting *caf1*, *pla* and *inv* genes [33].

Following species identification, total DNA was extracted from fleas collected in the field using Blood and Tissue Qiagen kit (Qiagen, Germany) and following manufacturer’s instructions. PCR technique was used to detect *Y. pestis* DNA on fleas using published protocol. Targeted genes were *pla*, *caf*1 and *inv*.

#### Serological screening to detect exposure to *Y. pestis.*

Serum samples or blood spots from each animal were tested for the presence of IgG antibodies against *Y. pestis* F1 antigen using Enzyme-linked immunosorbent assay (ELISA) [34,35].

## Results

### Small mammal capture

**Fig 2:**
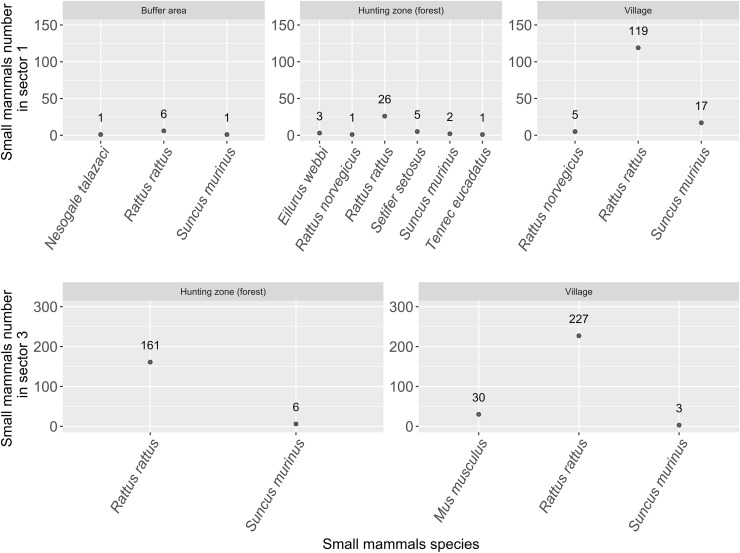
Distribution and specific diversity of small mammals in the sector 1 and sector 3 sites.

A total of 614 individuals of small mammals belonging to eight species were captured at the 22 sites of the two sectors (1 and 3) (Fig 2), after a total of 4,880 trap-nights (Table 1). Introduced mammals were represented by four species namely *Mus musculus*, *Rattus rattus*, *R. norvegicus* and *Suncus murinus* while endemic species were composed of four species, *Setifer setosus*, *Eliurus webbi*, *Nesogale talazaci* and *Tenrec ecaudatus.* Average capture rate was 0.13%.

**Table 1:** Small mammals’ diversity into sector 1 and 3.

Order	Family	Species	Sector 1	Sector 3	Total
**Afrosoricida**	**Tenrecidae**	*Nesogale talazaci*	1	0	1
**Afrosoricida**	**Tenrecidae**	*Setifer setosus*	5	0	5
**Afrosoricida**	**Tenrecidae**	*Tenrec ecaudatus*	1	0	1
**Rodentia**	**Nesomyidae**	*Eliurus webbi*	3	0	3
**Rodentia**	**Muridae**	*Mus musculus*	0	30	30
**Rodentia**	**Muridae**	*Rattus norvegicus*	6	0	6
**Rodentia**	**Muridae**	*Rattus rattus*	151	388	539
**Eulipotyphla**	**Soricidae**	*Suncus murinus*	20	9	29
**Total**			**187**	**427**	**614**

In sector 1, a total of 187 individuals from seven species were captured during 3,080 trap-nights: *E. webbi*, *N. talazaci*, *R. rattus*, *R. norvegicus*, *S. setosus*, *S. murinus* and *T. ecaudatus. Rattus rattus* accounted for 80.7% (151/187) of captured individuals and was found in all three habitats—villages, hunting zones and buffer zones—but was most frequent in villages followed by hunting zones. *Suncus murinus* were also found in all three habitats and more frequent in villages. It contributed for 10.6% (20/187) of captured individuals. Endemic species represented only 5.3% (10/187) of captured individuals and, in sector 1, they were only captured in the two types of forested habitats: buffer zones and hunting zones (Fig 2).

In sector 3, 427 individuals of three introduced species (*R. rattus*, *M. musculus and S. murinus*) were captured during 1,800 trap-nights. *Rattus rattus* accounted for 91% (388/427) of total capture. *Mus musculus* was only found inside houses and represented 7% (30/427) of the capture. Finally, S*uncus murinus* (9 individuals) was captured in the forest (n = 6) and inside houses (n = 3). Overall, the majority of small mammals (60.9%) were captured inside houses in sector 3 (Fig 2).

### Flea collection

A total of 1,754 fleas were collected from small mammals in sector 1 and 3. The global flea index recorded was 2.8. *Xenopsylla brasiliensis* was the most collected species (65.0%; n = 1,139), followed by *X. cheopis* (29.7%; n = 521), *Echidnophaga gallinacea* (4.6%; n = 80), *Synopsyllus fonquerniei* (0.6%; n = 11) and *Ctenocephalides felis* (0.1%; n = 2). One individual of *Xenopsylla* showed damaged body and could not be identified at species level (Table 2).

**Table 2:** Numbers and diversity of flea species collected in the study.

Sector	Locality	*Echidnophaga gallinacea*	*Xenopsylla cheopis*	*Ctenocephalides felis*	*Xenopsylla sp.*	*Xenopsylla brasiliensis*	*Synopsyllus fonquerniei*	*Total*
1	Ambalamahogo	0	20	0	0	0	2	22
1	Ambalarano	0	0	0	0	0	0	0
1	Ambodivoangy	0	0	0	0	0	0	0
1	Andaparaty	9	8	0	0	0	0	17
1	Ankiatombaka	0	0	0	0	0	0	0
1	Maitsiarongana	0	0	0	0	0	1	1
3	Andilambe	13	60	0	0	306	6	385
3	Andranonkazo	38	433	1	1	206	2	681
3	Tsarabanjina	20	0	1	0	627	0	648
**Total**		**80**	**521**	**2**	**1**	**1,139**	**11**	**1,754**

**Fig 3:**
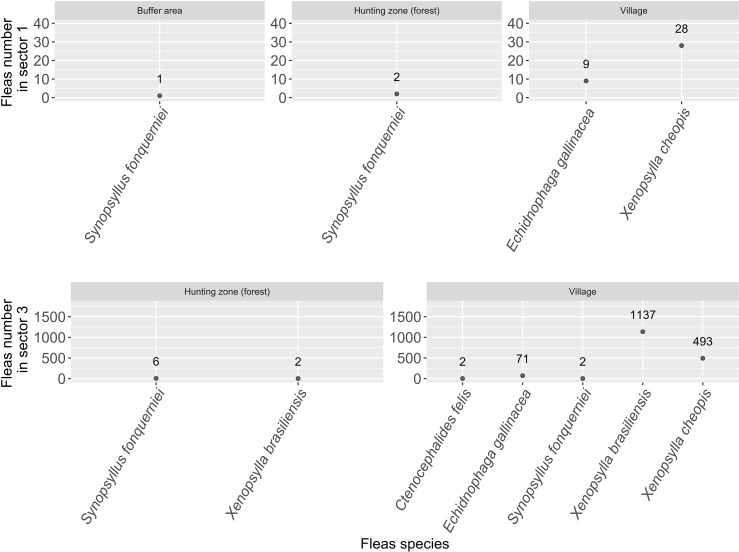
Distribution and specific diversity of fleas in sector 1 and sector 3 sites.

In sector 1, 19 small mammals allowed collecting 40 individual fleas which belonged to three species: *Xenopsylla cheopis* (n = 28; 70.0%), *Echidnophaga gallinacea* (n = 9; 22.5%) and *Synopsyllus fonquerniei* (n = 3; 7.5%). *Synopsyllus fonquerniei* individuals were all collected on *Rattus rattus* captured outside houses (i.e., on rats captured inside traps installed along transect line in hunting zone). The mean infestation rate was 0.21 fleas per small mammal (Fig 3).

In sector 3, 1,714 individual fleas composed of five species were collected on 205 small mammal individuals. *Xenopsylla brasiliensis* accounted for 66.5% of fleas (n = 1,139), *X. cheopis* for 28.7% (n = 493), *Echidnophaga gallinacea* for 4.1% (n = 71), *Synopsyllus fonquerniei* for 0.5% (n = 8) and *Ctenocephalides felis* for 0.1% (n = 2). One unidentified *Xenopsylla* sp. (0.1%) was also collected (Fig 3). The mean infestation rate was 4.01 fleas per small mammal.

### Bacteriological screening of *Y. pestis* in small mammals

All the 613 spleen samples collected from small mammals tested negative through TDR and bacteriology.

### Molecular screening of *Y. pestis* in small mammals and their fleas

All the 614 individual small mammals tested negative for *Y. pestis* using molecular diagnostic. However, among the 104 flea samples (individuals and pools) tested for the presence of *Y. pestis*, one individual of *Ctenocephalides felis* collected on *Rattus rattus* from Andranonkazo (sector 3) was found positive for *Y. pestis*.

### Serological screening of *Y. pestis* in small mammals

Of the 614 mammal serum samples tested, a single individual of *R. rattus* captured in Andilambe (sector 3) was found seropositive, indicating previous exposure of the individual to *Yersinia pestis* (Table 3).

**Table 3:** Serological screening of small mammals from Makira region.

Makira Region	Locality	Species	No. small mammals captured	No. of seropositive individuals (%)
Sector 1	Ambalamahogo			
		*Rattus rattus*	48	**0**
		*Setifer setosus*	3	**0**
		*Suncus murinus*	7	**0**
		*Tenrec ecaudatus*	1	**0**
	Ambalarano			
		*Eliurus webbi*	2	**0**
		*Rattus norvegicus*	2	**0**
		*Rattus rattus*	27	**0**
		*Suncus murinus*	2	**0**
	Ambodivoangy			
		*Rattus norvegicus*	4	**0**
		*Rattus rattus*	23	**0**
		*Setifer setosus*	2	**0**
		*Suncus murinus*	1	**0**
	Andaparaty			
		*Eliurus webbi*	1	**0**
		*Rattus rattus*	22	**0**
		*Suncus murinus*	1	**0**
	Ankiatombaka			
		*Rattus rattus*	10	**0**
		*Suncus murinus*	5	**0**
	Maitsiarongana			
		*Nesogale talazaci*	1	**0**
		*Rattus rattus*	21	**0**
		*Suncus murinus*	4	**0**
Sector 3	Andilambe			
		*Mus musculus*	8	**0**
		*Rattus rattus*	139	**(1) 0.7**
		*Suncus murinus*	7	**0**
	Andranonkazo			
		*Mus musculus*	10	**0**
		*Rattus rattus*	133	**0**
		*Suncus murinus*	1	**0**
	Tsarabanjina			
		*Mus musculus*	12	**0**
		*Rattus rattus*	116	**0**
		*Suncus. murinus*	1	**0**
	**Total**		**614**	**0.2**

## Discussion

The aim of our work was to investigate the circulation of *Y. pestis* in an area of northeastern Madagascar (Makira National Park), where plague outbreaks were suspected but scientific information was not available prior to this study. Our investigation concluded that the diversity of small mammal and flea populations in the area was important and compatible with the circulation of plague in the Makira region.

A total of eight species of small mammals were recorded at the different sites investigated in sectors 1 and 3 of the MNP region. The diversity of small mammals trapped in our study was lower than in previous studies conducted in Makira region, where 17 species had been recorded. That earlier study primarily sampled within the forest and used an additional trapping method—pitfall traps [36]. In our study, sampling efforts were primarily focused in zones close to human activities in order to assess the potential role of these animals in the maintenance and transmission of plague at these interfaces.

Strong differences in variety of small mammals, distribution of flea species and infestation rates were observed between the two sectors. In sector 1, both introduced and endemic species were represented, whereas in sector 3, only introduced small mammal species were captured. In sector 1, the infestation rate was more than 18 times lower than in sector 3. In sector 1, the majority of fleas captured in the village were *X. cheopis*, the main vector of plague in Madagascar, whereas *X. brasiliensis* was only collected in sector 3, where it was by far the most frequently collected species.

The black rat, *Rattus rattus,* represented the majority of the species captured. This species is known to be able to colonize different habitats throughout Madagascar, from anthropized to forested zones [28]. Scobie *et al.* [37] reported that black rats pose a serious threat to food security and public health in Madagascar, where they are a major cause of pre- and post-harvest crop losses and an important reservoir for many zoonotic diseases, including plague. In this study, a high rate of *R. rattus* was detected probably as a result of an adaptation of synanthropic rodents to human modified habitats. Similarly, the house mouse, *M. musculus,* is well known to occur inside houses in Madagascar [28] and was generally found inside houses in sector 3 during this survey. A previous study conducted in the Mandritsara district (sector 3), close to villages, also detected a very high percentage (93.3%) of *R. rattus*, followed by 5.6% of *M. musculus* and only 1.1% of house shrews, *S. murinus* [14] among small mammals captured.

Although tenrecs are cited as being a very commonly consumed species in this region [38], our study only managed to capture one individual of *T. ecaudatus*. This suggests that the capture method implemented might not be the most efficient approach to collect and sample tenrec species and other protocols should be considered.

Regarding flea diversity in an around Makira Natural Park, five species of fleas were found infesting small mammals, two of these (*X. cheopis* and *S. fonquerniei*) are known to be plague vectors in Madagascar [2] and were found in the two investigated sectors. Average flea index detected in our study (2.5) was close to the average index identified in other areas of Madagascar where plague outbreaks occur regularly, yet it varied greatly between sectors, (0.00 in Ambalamahogo, Ambodivoahangy and Ankiatombaka, 5.03 in Tsarabanjina). Although this index is considered a poor predictor of plague if *Y. pestis* is not detected, it is informative for implementation of vector control [39]. Here, the combination of a high flea infestation rate and the proof of exposure in one rat and the infection of one flea in sector 3 indicates that the risk of plague should not be overseen: awareness of communities and of local healthcare workers should be assessed and raised if needed, and rapid response teams should be trained if this is not yet the case.

This study expanded the known geographic distribution of the endemic plague vector *S. fonquerniei* in this lowland northern region of Madagascar (altitude less than 800 m), as this flea was previously mainly reported in the central highlands, above 800 m and at low temperatures [2,40]. It is important therefore to further investigate the distribution of this species for monitoring and control of plague. In the Central Highlands, *S. fonquerniei* is found mainly on rats caught outdoors as well as in forested areas [41,42] and shows a clear seasonal cycle, thriving in the middle and at the end of the dry and cold season [42]. The distribution and dynamics of this species in the lowlands need further assessment.

A high proportion of *Xenopsylla cheopis* was observed in the villages, especially inside houses as already reported in previous studies [42]. This cosmopolitan species is common in small mammals captured inside houses.

*Xenopsylla brasiliensis* is a well-known flea of rats in Africa and the southwestern Indian Ocean islands [43–45]. While *X. brasiliensis* is known as plague vector in other African countries such as Zimbabwe [43,44], no information is available regarding their potential role as vector of plague in Madagascar. However, we found a particularly high number of *X. brasiliensis* in *Rattus rattus* from Mandritsara district (sector 3). The ecology and distribution of this flea in Madagascar is poorly understood, as they have only recently been found in this northern region of Madagascar in a plague focus [14]. The very high infestation rate observed here and its known vector competence in other African settings pleads for further investigations to understand its geographical distribution and dynamics in the country to better assess its involvement in the circulation and maintenance of plague throughout the Island.

It is well known that *R. rattus* contributes significantly to the maintenance and transmission of plague in Madagascar [2,46]. In this study, the detection of a single seropositive individual of *R. rattus* in Andilambe provided evidence of the circulation of *Y. pestis,* although at very low level. In fact, reported seroprevalence of plague in small mammals is often quite low. Previous observations during a longitudinal murine surveillance in plague endemic foci reported seropositivity rates lower than 1% in black rat populations after two years of capture [47].

The finding of natural infection with *Y. pestis* in a cat flea, *C. felis*, is interesting as it is the first time that this flea species is found infected by *Y. pestis* in Madagascar. However, cat fleas have been reported as a competent vectors in eastern Africa, though with a lower efficiency compared to *Xenopsylla cheopis* [48]. In this region, the cat flea is commonly found inside houses but in the MNP region it was the least abundant among collected species (0.1% of collected fleas), only found in two sites out of sixteen (Andranonkazo and Tsarabanjina).

Our study identified the presence of the two known main plague vectors in Madagascar, *X. cheopis* and *S. fonquerniei*, in both investigated sectors in the northeastern part of the country. The two other species, *X. brasiliensis* and *C. felis,* also deserve attention as the first is a known plague vector in other African countries [49] and was particularly abundant in Sector 3 whereas the second which was found infected by *Y. pestis,* is acknowledged to be a competent vector of plague [48]. These findings suggest that the transmission cycle of *Yersinia pestis* in the northern portion of Madagascar is complex with the presence of four flea species and warrant further investigations and surveillance.

Reduction or discontinuance of surveillance and control, as well as poverty and poor sanitation are the main factors in the emergence and re-emergence of human plague cases, resulting from increased contacts with infected rodents and fleas [50]. Our study confirmed *Y.* *pestis* circulates at a low level in the area, and supports the reports of suspect human plague cases. The lack of investigation of these suspected cases suggests the need to strengthen murine and flea surveillance, the training of local health care and community agents and finally intervention capacity of response teams. It is therefore important to increase awareness and implement further surveillance strategies in wildlife and humans to better understand the circulation and significance of this pathogen in the poorly-resourced MNP region. Additionally, the concurrent testing of these potential rodent reservoirs for other endemic zoonotic pathogens such as *Bartonella*, *Rickettsia* and *Leptospira*, would be valuable to expand our knowledge on their zoonotic potential and improve infectious disease control strategies in an area where people have regular, close contact with the wildlife. Such investigations would also help assess the possibility of co-transmission with *Yersinia pestis*.

To propose sustainable surveillance and prevention measures to reduce the risk of plague in the Makira region, we need to consider host and environmental factors in disease ecology, and the susceptibility of fleas to vector control strategies, namely insecticide use. The resistance of certain wild mammal species to clinical expression of the disease, enables them to act as a reservoir which allows the maintenance of the pathogen. Insecticide resistance of fleas enables increased propagation of disease within the rodent population and more frequent transmission to humans [3].

One Health surveillance focuses on the relationship between human activities, the health of domestic, peri-domestic and wild animals and their environment. This association is highly relevant in the context of zoonotic disease surveillance especially in the MNP where local communities live in close proximity with wildlife, particularly synanthropic species and where ecosystems are rapidly changing. Plague is a zoonosis, that circulates within a complex system of ecological interactions between the pathogen, *Y. pestis*, its hosts, its vectors and the spatiotemporal variations of its ecosystems [51]. As already stated above, the black rat, a key plague reservoir highly adaptable to a variety of environments, represented the main species captured in this study, in both types of habitats studied (forests and villages) and both sectors. Environment changes (i.e., climatic changes, deforestation, urbanization) can induce changes in flea and rodent populations through the extension of rodent reservoir habitats (for example by replacing forests by steppes or farmlands) and modifications in population dynamics (possible outbreaks due to an increase in availability of food resources), but also, via the emergence of new vectors, reservoirs and new *Y. pestis* genotypes [50]. Alderson *et al*. [3] proposed that “To understand how climate phenomena may affect plague incidence, the effects of weather, temperature and precipitation on host, vector and bacterium, should be examined”.

Finally, surveillance benefits populations only if subsequent public health interventions and access to treatment are ensured. Limited healthcare access near natural parks in rural Madagascar is a significant barrier, as Evans *et al.* showed in the district of Ifanadiana (Vatovavy region) where consultation rates dropped by 28% for every additional kilometer between patients and community health workers [52]. In areas so remote and so heavily reliant on non-motorized transportation as MNP, improving access to healthcare is a critical challenge that demands urgent assessment and action.

## Conclusion

In conclusion, the diversity and abundance of small mammals and mainly invasive rodents and fleas detected combined with the confirmation of *Y. pestis* DNA in one specimen of *C. felis* collected on a *R. rattus* as well as the presence of one plague seropositive *R. rattus* provide evidence of *Y. pestis* circulation in the MNP region at low level and suggest that the area provides a suitable habitat for multiple plague reservoirs and vectors, posing a risk for the emergence of disease outbreaks in the local populations. Assessing knowledge, attitudes and practices towards bubonic plague in local communities and among healthcare workers would enable to identify key messages and populations to target in the context of awareness campaigns and strategic planning to improve surveillance activities. Leveraging plague surveillance efforts in rodent hosts would support improved monitoring and mitigation of human health threats in resource-limited settings such as the MNP where people have close contacts with wildlife and where limited knowledge on circulating pathogens is available.

## Supporting information

S1 Data Overview of the data associated with this study. (XLSX)
